# Changes in *Chlamydia trachomatis* risk before and after union formation and separation among women of reproductive age

**DOI:** 10.1093/eurpub/ckae074

**Published:** 2024-04-19

**Authors:** Niina S Metsä-Simola, Elina K Einiö, Pekka T Martikainen

**Affiliations:** Helsinki Institute for Demography and Population Health, University of Helsinki, Helsinki, Finland; Max Planck—University of Helsinki Center for Social Inequalities in Population Health, Helsinki, Finland; Helsinki Institute for Demography and Population Health, University of Helsinki, Helsinki, Finland; Max Planck—University of Helsinki Center for Social Inequalities in Population Health, Helsinki, Finland; Helsinki Institute for Demography and Population Health, University of Helsinki, Helsinki, Finland; Max Planck—University of Helsinki Center for Social Inequalities in Population Health, Helsinki, Finland; Max Planck Institute for Demographic Research, Laboratory of Population Health, Rostock, Germany

## Abstract

**Background:**

Not having an established relationship is associated with an elevated risk of *Chlamydia trachomatis* (CT) infection, but this might reflect selection into and out of unions. Although union formation and union separation are common events in reproductive age, little is known about changes in CT risk before and after these transitions.

**Methods:**

We linked Finnish Population Register data to the National Register of Infectious Diseases and used fixed-effects linear probability models that account for all time-invariant confounders to examine changes in women’s 6-month CT risk 3 years before and 3 years after entry into first cohabitation (*n* = 293 554), non-marital separation (*n* = 201 647) or marital separation (*n* = 92 232) during 2005–14.

**Results:**

From 3 years to 1 year before first union formation, the 6-month risk of CT increased slightly, peaking at 1.27% immediately prior to union formation (95% confidence interval 1.22–1.31). It declined sharply following union formation, being only 0.40% (0.34–0.46) 6–12 months after union formation with little changes thereafter. Among women separating from non-marital unions, the risk increased from 0.50% (0.42–0.57) to 1.45% (1.40–1.49) around the time of separation and decreased following separation. The pattern of findings was relatively similar for marital separation, although the observed risks and changes were smaller in magnitude.

**Conclusions:**

Our results based on longitudinal data and individual fixed-effects models indicate that the period immediately after separation may be causally associated with an elevated risk of CT. This suggests that recently separated women should be identified as a high-risk group for CT.

## Introduction

*Chlamydia trachomatis* (CT) is a major sexually transmitted infection (STI) both in Europe and the USA.^1–4^ In women, CT may lead to pelvic inflammatory disease, ectopic pregnancies and even infertility.^5^ With most cases diagnosed among adolescents and young adults,^2^^,^^5–8^ CT may have a large effect on women’s reproductive health over their entire reproductive age.

Previous cross-sectional studies have shown that those who are unmarried have a higher risk of CT than those who are currently married^1^^,^^5–7^^,^^9^ or cohabiting.^1^^,^^7^ However, these findings from cross-sectional studies may partly reflect selection into and out of unions, and it is still unclear how the risk of CT changes before and after union formation or separation. Although previous research has longitudinally followed individuals to establish associations between CT and later medical outcomes such as repeated CT infections^10^ or cervical cancer,^11^ to our knowledge, there are no longitudinal studies that assess how the risk of CT changes with changing union status in the general population.

Having a new partner has been shown to increase the risk of CT,^9^^,^^12^^,^^13^ and the time before forming a union may thus be particularly risky for getting infected. Young individuals are more likely to enter a non-marital cohabiting union than marriage, and in one study on British women of reproductive age, non-marital unions were associated with a higher CT risk than marriage.^7^ Similarly, an American study of 14- to 39-year-olds showed that women who lived with a non-marital partner had a somewhat higher prevalence of CT compared to those who were married.^1^ The finding could be explained, however, by age differences between the groups. Another period of elevated risk may occur around the time of union separation. A longitudinal survey study from Australia showed that young women who were recently divorced or separated were more likely to report an STI than other women,^14^ which might be because of their higher probability of initiating new sexual relationships.^15^ Separation is also a stressful event and may lead to high-risk behavior,^16^ which in turn could increase the risk of STIs.^3^^,^^17^

In the present study, we use linked register-based data on all Finnish women of reproductive age to examine changes in CT risk before and after union formation and union separation. In Finland, sexually active residents are encouraged to test for CT and other STIs whenever they switch partners, and screening for CT is commonly offered to young adults seeking contraceptive counseling. All pregnant women are screened by the end of the first trimester following national recommendations. In the public sector, both the testing and treatment of CT are free of charge. This is guaranteed by the infectious disease legislation (1227/2016) that also requires all healthcare laboratories to report patients with CT to a national register for monitoring infectious disease trends.^18^ The register linkage offers an internationally unique opportunity to examine changes in the risk of CT over the transition to union formation or separation without bias from self-reporting, non-response, or attrition.

We follow women for CT incidence before and after the date of entry into and exit out of marital and non-marital cohabitation and analyze changes in their risk of CT. Because unobserved characteristics of women may increase both the risk of union transitions and CT, we estimate individual fixed-effects (FE) models. These models adjust for all time-invariant observed and unobserved confounders such as personality, family background, and previous life experiences, and thus allow for stronger causal inference.^19^ To our knowledge, this is the first longitudinal study to examine changes in CT risk before and after union transitions.

## Methods

We used total population Finnish register data on all women aged 15–49 who moved in or out of marital or non-marital cohabitation from 2005 to 2014. Statistics Finland provided information on the exact dates of moving into (referred to herein as union formation) and moving out of marital and non-marital cohabitation (referred to herein as union separation), as well as annual socio-demographic information. These data were linked to the Finnish National Infectious Diseases Register (NIDR) maintained by the Finnish Institute for Health and Welfare using personal identification codes. In Finland, national surveillance of CT is based on mandatory notification of microbiologically confirmed cases to the NIDR under the Communicable Diseases Act.^18^ The study was conducted with permission from the Ethics Committee of Statistics Finland (TK/32/07.03.00/2020) and Findata (THL/3154/14.06.00/2022). The use of Finnish register data for purposes of scientific research carried out in the public interest does not require informed consent from participants.

We included women who formed their first union (*n* = 293 554) and followed them for CT incidence 3 years before the exact date of union formation. Because the vast majority of first unions were non-marital when living together started (94.2%), we did not differentiate between marital and non-marital union formation. The follow-up for CT continued 3 years after the date of union formation, unless the woman was censored earlier due to death or separation (*n* = 88 971). For separating women, we included the first non-marital (*n* = 201 647) or marital (*n* = 92 232) separation observed between 2005 and 2014. Separating women were followed for CT up to 3 years before the exact date of separation. Women separating from unions <3 years (*n* = 180 649) entered the follow-up at the time of union formation. After separation, women were followed for three more years, unless they formed a new union or died, in which case they were censored in that observation interval (*n* = 101 140). Dissolving marriages lasted 12–13 years on average, and non-marital cohabiting unions lasted approximately 3 years.

The follow-up time before and after union transitions was divided into 6-month intervals. We examined changes in CT incidence between these intervals using linear probability models and applied an individual FE estimator. The FE estimator uses individuals as their own controls, effectively controlling for time-invariant heterogeneity such as differences in personality, socio-economic and family background, and life experiences that are likely to affect both union transitions and CT risk.^19^ The models were adjusted for annually measured age and calendar time.

## Results

Between 2005 and 2014, we observed 293 554 women of reproductive age forming their first union (table 1). During the 6-year follow-up period, 8.0% of them were infected with CT. The women were relatively young with a mean age of 22.8 years, and two-thirds of the women did not (yet) have a secondary or tertiary education at the time of union formation. The 201 647 women separating from non-marital unions were younger (mean age 27.8 years) than women separating from marital unions (mean age 37.1 years), and they were much more likely to be diagnosed with CT (7.2% vs. 3.1% diagnosed at least once during follow-up). Their average follow-up time was also somewhat shorter, reflecting the shorter average duration of non-marital unions. Separating women were more highly educated than women who formed their first union, given that they were older, and most were separating from their first union.

**Table 1:** Characteristics of Finnish women of reproductive age who started to cohabit for the first time or separated from non-marital or marital cohabitation between 2005 and 2014

	Start of first cohabitation (*N* = 293 554)	Separation from non-marital cohabitation (*N* = 201 647)	Separation from marital cohabitation (*N* = 92 232)
Mean (SD) age at union transition, years	22.8 (5.1)	27.8 (8.1)	37.1 (7.4)
Calendar year, mean (SD)	2009.3 (2.9)	2009.2 (2.9)	2009.2 (2.8)
Education, %			
Basic or less	68.4	16.5	16.9
Intermediate (ISCED 3–4)	24.2	59.0	41.8
Tertiary (ISCED 5–8)	7.5	24.5	41.3
Average follow-up time, years	5.6	4.6	5.5
Proportion of women infected during follow-up, %	8.0	7.2	3.1

**Figure 1:**
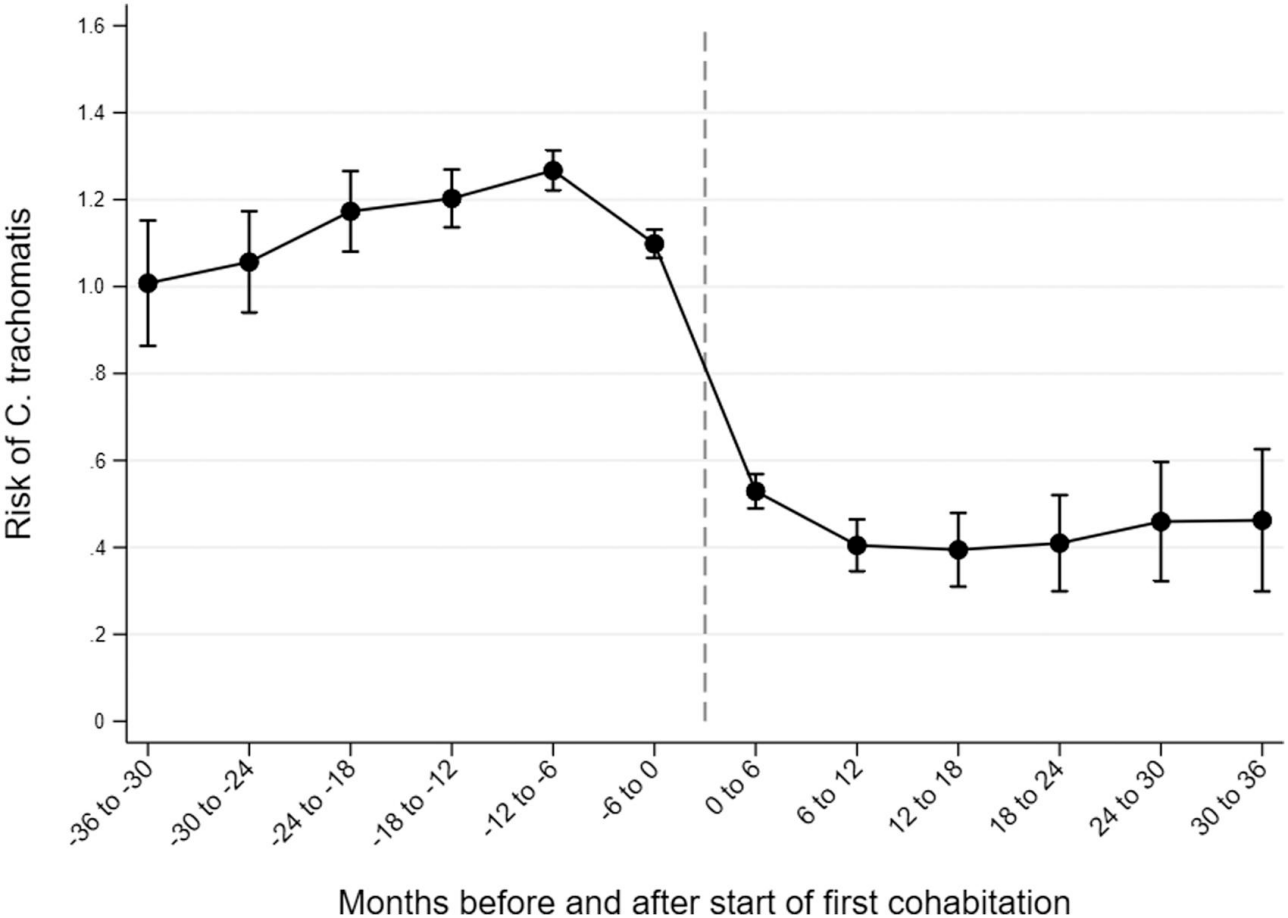
Changes in the 6-month risk of *Chlamydia trachomatis* infection 3 years before and 3 years after the start of first cohabitation among Finnish women of reproductive age.

In the individual FE models, women’s risk of being diagnosed with CT clearly declined at the time of first union formation (figure 1, Supplementary table S1). While women’s 6-month CT risk increased slightly from 3 years to 6 months before union formation, reaching 1.27% (95% confidence interval 1.22–1.31) during the time interval 6–12 months before union entry, it declined to 1.10% (1.07–1.13) immediately before union formation, and then more rapidly declined to 0.53% (0.49–0.57) during the 6 months immediately after the start of the union. During the time interval 6–12 months after union formation, CT risk had declined to 0.40% (0.34–0.46), after which it remained similar until the end of the 3-year follow-up.

**Figure 2:**
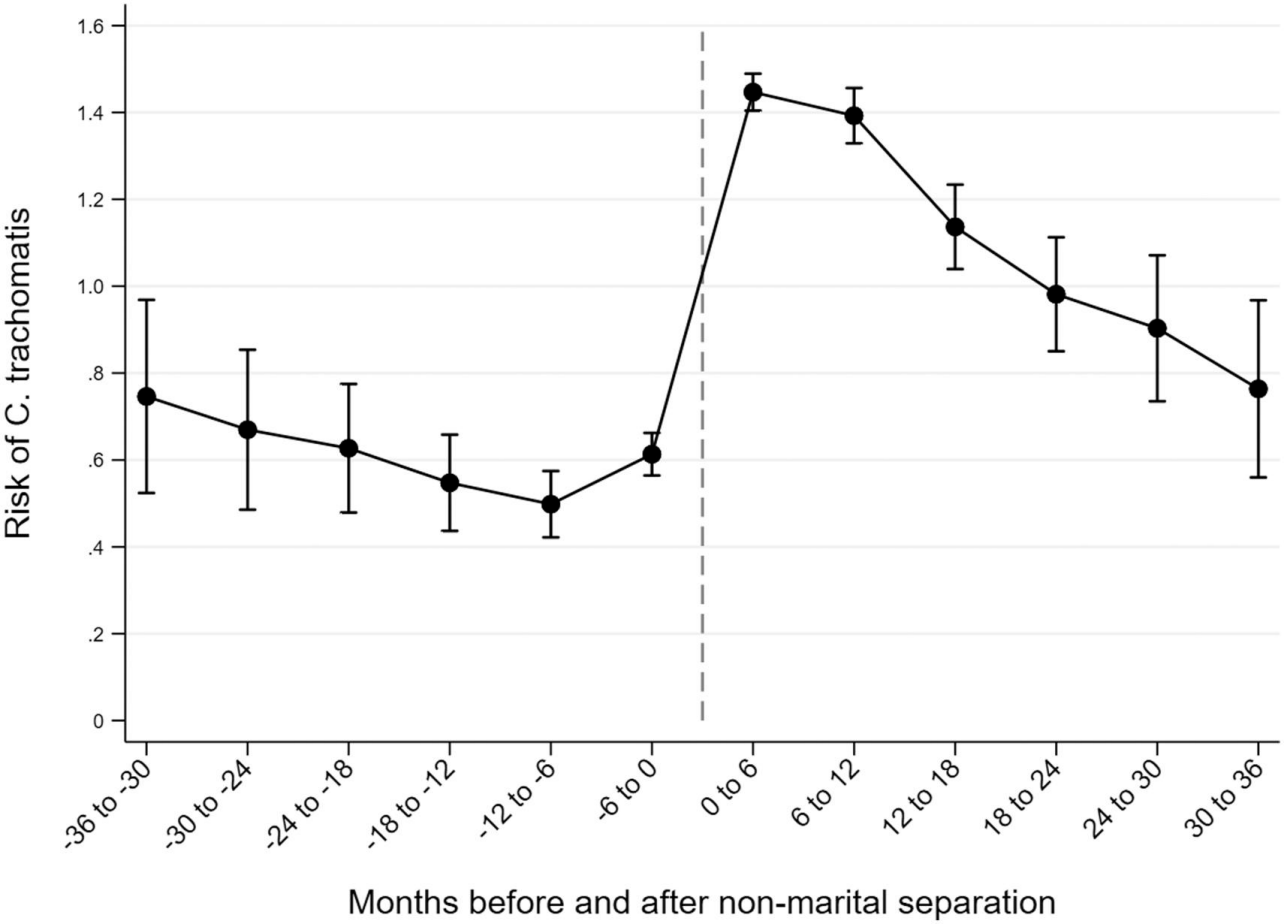
Changes in the 6-month risk of *Chlamydia trachomatis* infection 3 years before and 3 years after non-marital separation among Finnish women of reproductive age.

At the time of non-marital separation, women’s risk of CT increased considerably (figure 2, Supplementary table S2). There was first a slight decline in CT risk from 3 years to 6 months before the date of separation, so that during the time interval of 6–12 months before separation, the risk of CT was 0.50% (0.43–0.57). The risk then increased to 0.61% (0.56–0.66) during the 6 months immediately before separation, followed by a much more rapid increase to 1.45% (1.40–1.49) during the 6 months immediately after separation. The risk remained high 6–12 months after separation, but thereafter a constant decline was observed, with the 6-month risk of CT approaching the pre-separation level. Noteworthy, the risk of CT fell below the risk among women who had never been in a union before.

**Figure 3:**
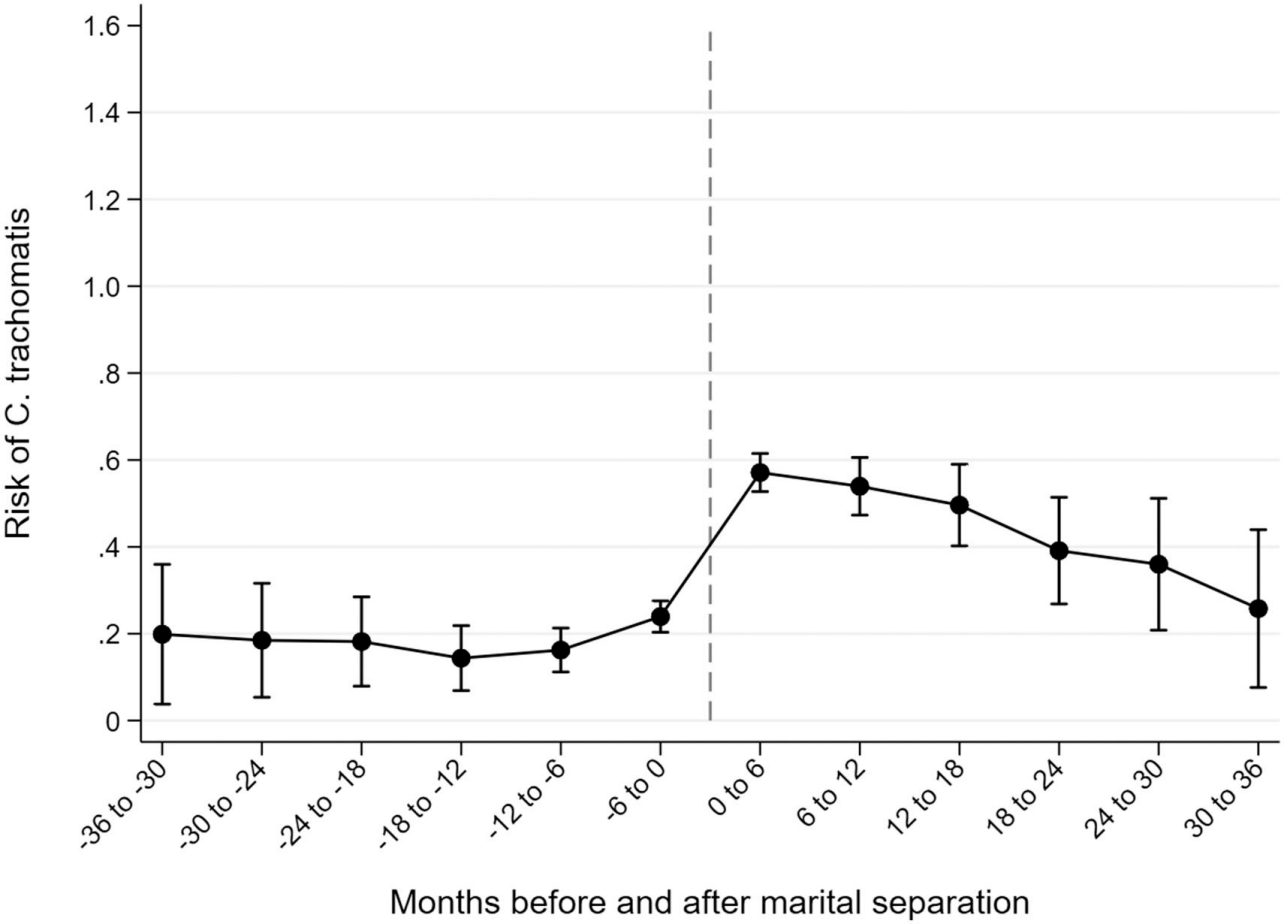
Changes in the 6-month risk of *Chlamydia trachomatis* infection 3 years before and 3 years after marital separation among Finnish women of reproductive age.

The risk of CT was clearly lowest among cohabiting women who were married (figure 3, Supplementary table S3). It remained at about 0.20% from 3 years to 6 months before separation and only increased to 0.24% (0.20–0.28) during the 6-month period immediately before the date of marital separation. Thereafter, an increase to 0.57% (0.53–0.61) immediately after separation was observed, followed by a steady decline during the 3-year post-separation follow-up.

## Discussion

In this study, we assessed changes in the risk of CT before and after union formation and union separation. Our main findings show that the risk of CT infection decreases following first union formation and increases following separation, especially shortly after separation from non-marital cohabitation as compared to separation from marriage. Our results from FE models are unique in that they are adjusted for all time-invariant confounders, such as personality, family and socio-economic characteristics. The association between union formation and a reduced CT risk observed in our study is thus not a result of women with a high CT risk remaining single and those with a lower CT risk forming unions. Although being in a union appears to be protective against CT, an increasing pattern of infections is observed before entry into the first union, when women are presumably looking for a partner. A decline in CT risk also continues during the first months after union formation. CT is often asymptomatic,^2^^,^^4^^,^^20^ and some previously infected women might thus be diagnosed once already in a union. It is also possible that some women are infected during the early months of their first union, either because their partner was previously infected or because they or their partner had sex with other partners after union formation.

Our results show that women have an increased risk of CT immediately following separation, particularly if the separation is non-marital. However, over the time since separation, CT risk steadily declines and falls below the level observed among women who have never been in a union before. There are several possible explanations for this time pattern of CT risk over time after separation. Recently separated women have been suggested to have a higher probability of initiating new sexual relationships, even as compared to other unmarried women,^15^ and having multiple partners has been identified as an independent risk factor for CT.^3^^,^^21^^,^^22^ As women form new stable unions over time and sexual activity with multiple partners declines, CT incidence also declines at longer durations of follow-up. However, this is probably not the only explanation, as in our analyses, women are censored at the time of starting to cohabit again. The high CT risk immediately after separation may also be related to the strain experienced during the separation process. Symptoms of poor mental health peak around the time of separation,^23–25^ and individuals in poor mental health are more likely than others to engage in unhealthy behaviors such as excessive alcohol use,^26^ which are associated with an increased risk of STIs.^3^^,^^17^ This suggests that support for separating women might not only benefit their mental health but also reduce their risk of CT.

Although many countries monitor the incidence of CT infections, our ability to link data on CT infections with information on union transitions made it possible to examine changes in CT risk before and after union formation and separation without self-report bias, non-response or attrition, which are all common problems in survey studies. While the large number of CT diagnoses among young women allowed a detailed estimation of the risk of CT surrounding union formation and separation from both non-marital and marital unions, this study is not without limitations. Because CT is often asymptomatic,^4^^,^^20^ diagnoses may be delayed. Although testing is free of charge and easily available in Finland, some individuals may choose to avoid or delay testing in the absence of symptoms. Instead of the probability of contracting CT, our estimates thus reflect the probability of receiving a diagnosis within a specific six-month interval. However, we have no reason to believe that this delay would systemically bias our results. We also lacked information about sexual partners and relationships without cohabitation, as well as other risk factors such as excessive alcohol use. Although our FE models effectively controlled for all measured and unmeasured time-invariant characteristics such as personality and family background, which are important predictors of many behavioral risks, future studies should explore the contribution of these factors directly in explaining the association between union transitions and CT risk.

Taken together, our findings show that in addition to the time before the first union formation, the time immediately after separation is associated with an elevated risk of CT, particularly after non-marital separation. The credibility of our results is significantly strengthened by the longitudinal repeated measurement around the time of union transitions and the analyses based on individual FE models that account for confounding based on time-invariant characteristics. The results suggest that women going through separation should be identified as a high-risk group that might benefit from support and targeted interventions. Possible future increases in non-marital cohabitation and union instability may lead to further increases in CT incidence among young adults in many high-income countries.

## Supplementary Material

ckae074_Supplementary_Data

## Data Availability

The data used in this study were collected by register authorities and are not publicly available. Those interested may apply for permission to use these data for scientific research from the register holders Statistics Finland and the Finnish Institute of Health and Welfare. Data from Statistics Finland: tutkijapalvelut@tilastokeskus.fi. Data from the Finnish Institute of Health and Welfare: info@findata.fi. Key points Women’s risk of *C. trachomatis* slightly increases before first union formation but declines considerably once the union begins. Large increases in *C. trachomatis* risk are observed when the union ends, particularly if the union was non-marital, but the risk steadily declines over time since separation. Recent separation should be acknowledged as a risk factor for *C. trachomatis* in women of reproductive age. Given the increasing union instability among young adults, the overall incidence of *C. trachomatis* could also rise.
