# Supplementary material for: Changes in Chlamydia trachomatis risk before and after union formation and separation among women of reproductive age
Source: Eur J Public Health. 2024 Apr 19;34(5):890–4. doi: 10.1093/eurpub/ckae074 (PMC11445784; doi:10.1093/eurpub/ckae074)
Supplement: ckae074_Supplementary_Data [file ckae074_supplementary_data.pdf]

Supplementary Table 1. Estimated six-month risk of *Chlamydia trachomatis* infection three years before and three years after the start of first cohabitation among Finnish women of reproductive age; individual fixed-effects models

Time interval in relation to start of cohabitation	Estimated proportion	95% confidence interval	
30-36 months before	1.01	0.86	1.15
24-30 months before	1.06	0.94	1.17
18-24 months before	1.17	1.08	1.27
12-18 months before	1.20	1.14	1.27
6-12 months before	1.27	1.22	1.31
0-6 months before	1.10	1.07	1.13
0-6 months after	0.53	0.49	0.57
6-12 months after	0.40	0.34	0.46
12-18 months after	0.39	0.31	0.48
18-24 months after	0.41	0.30	0.52
24-30 months after	0.46	0.32	0.60
30-36 months after	0.46	0.30	0.63

Supplementary Table 2. Estimated six-month risk of *Chlamydia trachomatis* infection three years before and three years after separation from non-marital cohabitation among Finnish women of reproductive age; individual fixed-effects models

Time interval in relation to non-marital separation	Estimated proportion	95% confidence interval	
30-36 months before	0.75	0.52	0.97
24-30 months before	0.67	0.49	0.85
18-24 months before	0.63	0.48	0.77
12-18 months before	0.55	0.44	0.66
6-12 months before	0.50	0.42	0.57
0-6 months before	0.61	0.56	0.66
0-6 months after	1.45	1.40	1.49
6-12 months after	1.39	1.33	1.46
12-18 months after	1.14	1.04	1.23
18-24 months after	0.98	0.85	1.11
24-30 months after	0.90	0.74	1.07
30-36 months after	0.76	0.56	0.97

Supplementary Table 3. Estimated six-month risk of *Chlamydia trachomatis* infection three years before and three years after separation from non-marital cohabitation among Finnish women of reproductive age; individual fixed-effects models

**Time interval in relation to marital separation**	**Estimated proportion**	**95% confidence interval**	
30-36 months before	0.20	0.04	0.36
24-30 months before	0.18	0.05	0.32
18-24 months before	0.18	0.08	0.28
12-18 months before	0.14	0.07	0.22
6-12 months before	0.16	0.11	0.21
0-6 months before	0.24	0.20	0.28
0-6 months after	0.57	0.53	0.61
6-12 months after	0.54	0.47	0.61
12-18 months after	0.50	0.40	0.59
18-24 months after	0.39	0.27	0.51
24-30 months after	0.36	0.21	0.51
30-36 months after	0.26	0.08	0.44
